# Zyxin Mediates Vascular Repair *via* Endothelial Migration Promoted by Forskolin in Mice

**DOI:** 10.3389/fphys.2021.741699

**Published:** 2021-10-08

**Authors:** Xuya Kang, Yanan Deng, Yang Cao, Yingqing Huo, Jincai Luo

**Affiliations:** Laboratory of Vascular Biology, Beijing Key Laboratory of Cardiometabolic Molecular Medicine, School of Future Technology, Institute of Molecular Medicine, Peking University, Beijing, China

**Keywords:** zyxin, vascular injury, re-endothelialization, endothelial migration, forskolin

## Abstract

**Background and Purpose:** Endothelial repair upon vascular injury is critical for the protection of vessel integrity and prevention of the development of vascular disorders, but the underlying mechanisms remain poorly understood. In this study, we investigated the role of zyxin and its associated cyclic adenosine monophosphate (cAMP) signaling in the regulation of re-endothelialization after vascular injury.

**Experimental Approach:** In *zyxin*-/- and wild-type mice, wire injury of the carotid artery was carried out, followed by Evans blue staining, to evaluate the re-endothelialization. Mice with endothelium-specific zyxin knockout were used to further determine its role. An *in vitro* wound-healing assay was performed in primary human endothelial cells (ECs) expressing zyxin-specific short-hairpin RNAs (shRNAs) or scrambled controls by measuring cell migration and proliferation. The effects of the cAMP signaling agonist forskolin were assessed.

**Key Results:** The re-endothelialization of the injured carotid artery was impaired in zyxin-deficient mice, whereas the rate of cell proliferation was comparable with that in wild-type controls. Furthermore, endothelium-specific deletion of zyxin led to similar phenotypes. Knockdown of zyxin by shRNAs in primary human ECs significantly reduced cell migration in the wound-healing assay. Notably, forskolin enhanced endothelial migration in a dose-dependent manner, and this was dependent on zyxin through its interaction with vasodilator-stimulated phosphoprotein. In addition, forskolin promoted the re-endothelialization of the injured carotid artery, and this was compromised by zyxin deficiency.

**Conclusion and Implications:** This study reveals zyxin as a new player in endothelial repair, which is promoted by forskolin, after vascular injury. Thus, zyxin-mediated signaling might be a potential treatment target for diseases involving vascular injury.

## Introduction

Normal endothelial cells possess antithrombotic and anti-inflammatory functions and maintain tissue perfusion and vascular tone. Thus, the integrity of the endothelial monolayer is essential for vascular homeostasis. It is known that endothelial cells (ECs) are prone to injury after exposure to both physical factors (such as disturbed blood flow or hypertensive stretch) and abnormal metabolic conditions (such as hyperglycemia or hyperlipidemia) (Landmesser et al., 2004). Endothelial injury is not only an early event in atherosclerotic vascular disease (Ross, 1999; Libby, 2000) but also a key element in recurrent in-stent restenosis after treatment of coronary stenosis (King et al., 2010). Rapid endothelial restoration, or re-endothelialization, is correlated with diminished neointima and plaque formation after injury (Versari et al., 2007). Although some available drugs have been shown to reduce vascular injury, their efficacy is insufficient. Therefore, new approaches that aim to promote endothelial repair are required for the effective prevention and treatment of atherosclerosis and in-stent restenosis. Despite extensive studies on the molecular mechanisms and signaling pathways that mediate endothelial injury; however, the regulatory mechanisms responsible for endothelial regeneration and vascular repair remain incompletely understood.

A cell moves by exerting forces from the cytoskeleton to the substrate *via* focal adhesion (FA), and then retracting and propelling the cell body forward (Parsons et al., 2010). In this study, we focused on zyxin, a well-known FA molecule. Zyxin is a phosphoprotein, which localizes to focal contacts, circumferential actin filament bundles, and stress fibers (Beckerle, 1986, 1997; Crawford et al., 1992). As a member of the Lin11, Isl- 1, and Mec-3 (LIM) domain protein family, zyxin has an extensive proline-rich NH2-terminal domain and three COOH-terminal copies of the LIM motif, interacting with α-actinin (Crawford et al., 1992) and vasodilator-stimulated phosphoprotein (VASP) (Reinhard et al., 1995). The cumulative body of evidence shows that zyxin is a key regulator of actin assembly and is implicated in the regulation of cell migration, growth, and differentiation *via* several signaling pathways. Recently, we showed for the first time that endothelial zyxin regulates thrombosis and protects vessels against bleeding injury *via* a cyclic adenosine 3,5-monophosphate-protein (cAMP)-protein kinase A (PKA) pathway (Han et al., 2017). In this study, we set out to study its role in re-endothelialization using a vessel injury model in mice and explored the therapeutic potential of zyxin signaling to accelerate endothelial recovery after vascular injury.

## Methods

### Experimental Animals

Ten-week-old male C57BL/6 mice were used for the wire injury studies. To check the endothelium-specific knockout, 6-week-old male mice were used for brain vascular endothelial cell isolation and western blot analysis. *Zyxin-/-* (ZYX KO) mice were purchased from Jackson Laboratory (Bar Harbor, ME, United States), knockout mice and wild-type (WT) littermates were produced by intercrossing heterozygous mice. *Tie 2-Cre* mice were kindly gifted by Professor Xiao Yang (Beijing Institute of Lifeomics, Beijing, China). *Zyx*^*flox*/*flox*^ mice were constructed by inserting Loxp into the introns after exons 5 and 8 of zyxin. About 9 kb of zyxin genomic DNA spanning from exons 6 to 8 was amplified by polymerase chain reaction (PCR), subcloned into pGEM-Teasy Vector (Promega, Madison, WI, United States), and confirmed by DNA sequencing. The targeting vector containing loxP sites in the zyxin intron was transfected into embryonic stem cells by electroporation. Homologous recombinant clones were identified by Southern blotting. Two independent clones were used to generate chimeras with C57BL/6 blastocysts, and the male chimeras were further bred with female C57BL/6 to get *Zyx*^*flox*/+^ heterozygotes. The genotype of the offspring was identified by PCR using primer pairs. The genotyping primers for *Zyx*^*flox*/*flox*^ mice were 5'- CATTGTGGACCAGGCCAATC-3' for forward, 5'- GCCAATGACAAGACGCTGG−3' for neo and 5'- GTCCACCATGGCAACAGAC−3' for reverse, floxed for 540 bp, and WT for 349 bp. The *Zyx*^*flox*/*flox*^ mice were interbred with *Tie 2-Cre* mice to generate endothelium-specific zyxin knockout (EZKO) mice (*Zyx*^*flox*/*flox*^; *Tie 2-Cre/*+); *Zyx*^*flox*/*flox*^; *Tie 2-Cre/*- mice were used as controls (CTR). The animals were maintained in the Center for Experimental Animals (a facility accredited by the Association for Assessment and Accreditation of Laboratory Animal Care) at Peking University, Beijing, China, under 12-h light/12-h dark cycle conditions with free access to food and water. All studies involving animals followed protocols approved by the Committee for Animal Research of Peking University and conformed to the Guide for the Care and Use of Laboratory Animals.

### Mouse Common Carotid Artery Wire Injury Model

Wire injury of the mouse common carotid artery was induced in the 10-week-old mice. The left common carotid artery (LCCA), left internal carotid artery (LICA), and left external carotid artery (LECA) were exposed through a ventral median incision in the neck, and carefully dissected free from surrounding nerves and fascia. Microvascular clips were placed around the proximal LCCA and LICA for temporary control of blood flow, while a 6-0 suture was placed around the LECA. A small incision was made in the LECA and then a.014-in guidewire (AGH14600; Asahi, Aichi, Japan) was advanced into the LCCA until 5 mm from the bifurcation, rotated five times, and then removed from the artery, which was tied off proximally. Subsequently, the microvascular clip was removed, and the skin incision was closed. To explore the effect of forskolin (FSK) on re-endothelialization, the mice were divided into two groups: a control group, receiving a daily intraperitoneal injection of vehicle (dimethyl sulfoxide, DMSO, in saline, 1:100 in volume), and an FSK group, receiving a daily intraperitoneal injection of FSK at 10 mg kg^−1^ (equivalent to 1 mg kg^−1^ in human doses) (Rios-Silva et al., 2014; Wang et al., 2019). The administration of the vehicle or FSK was initiated after wire injury and continued for 3 or 5 days.

### Evaluation of Re-endothelialization

Re-endothelialization was evaluated 0, 3, and 5 days after injury by left ventricle injection of 50 μl 5% Evans blue dye. The Evans blue-stained area was quantified by blinded image analysis.

### Common Carotid Artery Whole-Mount Staining

For common carotid artery whole-mount staining, the LCCA was cut open along the axis of the vessel flattened and fixed with 4% paraformaldehyde (PFA) at room temperature for 20 min. After that, the LCCA was permeabilized and blocked using 0.1% Triton X-100 with 1% horse serum and 2% bovine serum albumin (BSA) in phosphate-buffered saline (PBS) at room temperature for 1 h before incubation with primary antibodies to Pecam1 (rat, 1:500, 553370; RRID: AB_394816; BD Bioscience, San Jose, CA, United States) and anti Ki67 (rabbit, 1:500, ab15580, RRID: AB_443209; Abcam, Cambridge, United Kingdom) at 4°C overnight. Then, it was washed three times with PBS for 5 min each and incubated with fluorophore-conjugated secondary antibody at room temperature for 1 h. Finally, it was washed and incubated with 4', 6-diamidino-2 phenylindole dihydrochloride (DAPI) before mounting.

### Morphometry and Immunohistochemistry

OCT-embedded common carotid arteries were cut in 8-μl cross-sections. An analysis was carried out in the injured left common carotid artery, and the right one served as control. Neointima formation was analyzed by hematoxylin and eosin (HE) staining. For immunohistochemistry staining, the sections were stained with primary antibodies to zyxin (mouse, 1:500, H00007791-M01, RRID: AB_2221180; Abnova, Taipei, Taiwan) and anti Ki67 (rabbit, 1:500, ab15580, RRID: AB_443209; Abcam, Cambridge, United Kingdom), horseradish peroxidase-conjugated goat anti-mouse or anti-rabbit immunoglobulin G (IgG), and 3,3-diaminobenzidine, successively. The sections were then counterstained with hematoxylin.

### Cell Culture

Human umbilical vein endothelial cells were isolated from human umbilical veins using type I collagenase, and human amniotic epithelial cells (HAECs) were purchased from ATCC (CRL-4052, RRID: CVCL_Z065; Manassas, VA, United States). Mouse brain vascular endothelial cells (BECs) were isolated from mouse brain tissue using type II collagenase. The cells were cultured in M199 (31100035; Gibco, Amarillo, TX, United States) containing 20% fetal bovine serum (FBS) (Hyclone, Logan, UT, United States), recombinant human fibroblast growth factor (Sigma-Aldrich, St. Louis, MO, United States), and heparin (Sigma-Aldrich, St. Louis, MO, United States). Two hundred ninety-three T cells (ATCC, CRL-3216, RRID: CVCL_0063) were cultured in Dulbecco's Modified Eagle Medium (DMEM) containing 10% FBS. The cells were passaged by 0.05% trypsin digestion and maintained in a humidified incubator at 37°C with 5% CO_2_.

### Western Blot Analysis

For western blot analysis, the cells were washed twice with ice-cold PBS and lysed for 10 min in a cold radioimmunoprecipitation assay (RIPA) buffer (with protease inhibitor cocktail) on ice and then centrifuged at 14,000 rpm for 15 min. Proteins were resolved by 10% SDS-PAGE and transferred to hydrophilic polyvinylidene fluoride (PVDF) membranes, blocked with 5% nonfat milk in TBST for 1 h at room temperature, and then incubated with primary antibodies against zyxin (mouse, 1:500, H00007791-M01, RRID: AB_2221180; Abnova, Taipei, Taiwan) and Erk2 (rabbit, 1:1000, sc-292838, RRID: AB_2650548; Santa Cruz Biotechnology, Dallas, TX, United States) at 4°C overnight. The membranes were then washed four times for 10 min in TBST and then incubated with a second antibody at room temperature for 1 h. After three washes in TBST for 10 min, the membrane was assessed using an enhanced chemiluminescence western blotting detection kit.

### DNA Construction

Two shRNA constructs of zyxin were generated by the synthesis of double-strand oligos and ligation into the pLKO.1 vector with the targeting sequences 5'-CTGGGTCACAACCAAATCA-3' and 5'-GGCGACGAATTGACCAAAGCA-3'. A scrambled sequence was selected and served as a negative control. The zyxin VASP-binding mutant (4F>A mutant) was generated by the synthesis of double-strand oligos of 174 bp, which replaced the 211–213, 277–279, 310–312, and 340–342 bp segments of zyxin to change phenylalanine to alanine, and ligation into pBabe-puro (plasmid #1764-DNA.cg; Addgene, Watertown, MA, United States) vectors (Han et al., 2017; Li et al., 2018).

### Cell Transfection and Treatment

Preparations of lenti- or retrovirus were made in the 293 T cells. For transfection, the 293 T cells were seeded and grown for 24 h until 90% confluence was reached. Transfection complexes (polyethyleneimine and target and packaging plasmids) were formed at room temperature in a serum-free medium for 15 min before addition to the cells, followed by 6-h incubation and replacement with a complete medium for 48 h. Then, the virus-containing supernatants were harvested. For infection, virus-containing supernatant was mixed with a fresh medium, and polybrene was added. ECs were incubated in the virus-containing medium for 24 h. The medium was replaced with a culture medium after the infection.

### Cell Scratching Wound-Healing Assay

Cell migration was analyzed by a wound-healing assay. HUVECs and HAECs were cultured in 6-well plates until they reached 100% confluence. After that, the medium was changed to M199 with 6% FBS and left for 6 h. The cell layer was then scratched using a 200-μl pipette tip, and the cells were cultured in 6% FBS M199. During this assay, the forskolin was added for stimulation at different concentrations after scratching when needed. Proliferation inhibitor mitomycin C 5 μg/ml was used. Images of the cells were captured at baseline directly after scratching, and 16 and 24 h later under an inverted microscope (IX73; Olympus, Tokyo, Japan).

### Cell Immunofluorescence Staining

Briefly, the HUVECs were washed, fixed, and permeabilized with 4% formaldehyde and 0.1% Triton-X 100 for 10 min at room temperature. The primary antibody for Ki67 (rabbit, 1:500, ab15580, RRID: AB_443209; Abcam, Cambridge, United Kingdom) was incubated for 1 h at room temperature. Then, it was washed and incubated with a fluorophore-conjugated secondary antibody at room temperature for 1 h. Finally, the cells were washed and incubated with DAPI before mounting.

### Materials and Reagents

The following chemicals, unless specifically indicated, were from Sigma-Aldrich (St. Louis, MO, United States): forskolin (F6886), epinephrine (E4642), heparin (H3149), 4', 6-diamidino-2 phenylindole dihydrochloride (DAPI, D8417), mitomycin C (M5353), and dimethylsulfoxide (D2650). Anti ERK2 (rabbit, 1:1000, sc-292838, RRID: AB_2650548; Santa Cruz Biotechnology, Dallas, TX, United States) and anti-zyxin (mouse, 1:500, H00007791-M01, RRID: AB_2221180; Abnova, Taipei, Taiwan) were used for western blot. Anti Pecam1 (rat, 1:500, 553370, RRID: AB_394816; BD Bioscience, San Jose, CA, United States) and anti Ki67 (rabbit, 1:500, ab15580, RRID: AB_443209; Abcam, Cambridge, MA, United States) were used for whole-mount staining. Anti-zyxin (mouse, 1:500, H00007791-M01, RRID: AB_2221180; Abnova, Taipei, Taiwan) and anti Ki67 (rabbit, 1:500, ab15580, RRID: AB_443209; Abcam, Cambridge, United Kingdom) were used for immunohistochemistry and immunofluorescence staining.

### Data and Statistical Analysis

For all statistically analyzed studies, experiments were performed five times independently. The experiments were carried out in a randomized manner. The results are expressed as the mean ± SD. The number of samples in each group is shown in each figure. The data analyses were carried out in a blinded manner. Statistical differences between the two groups were analyzed by Student's *t*-test. The one-way or two-way ANOVA test was performed for multiple group comparisons, the level of significance was set at *P* < 0.05 (^*^), and the normality and homogeneity were tested before application. Concentrations of response curves were fitted in GraphPad Prism 7.0 (RRID: SCR_002798; GraphPad Software, San Diego, CA, United States).

## Results

### Zyxin Is Required for Vascular Recovery of Injured Mouse Arteries

**Figure 1:**
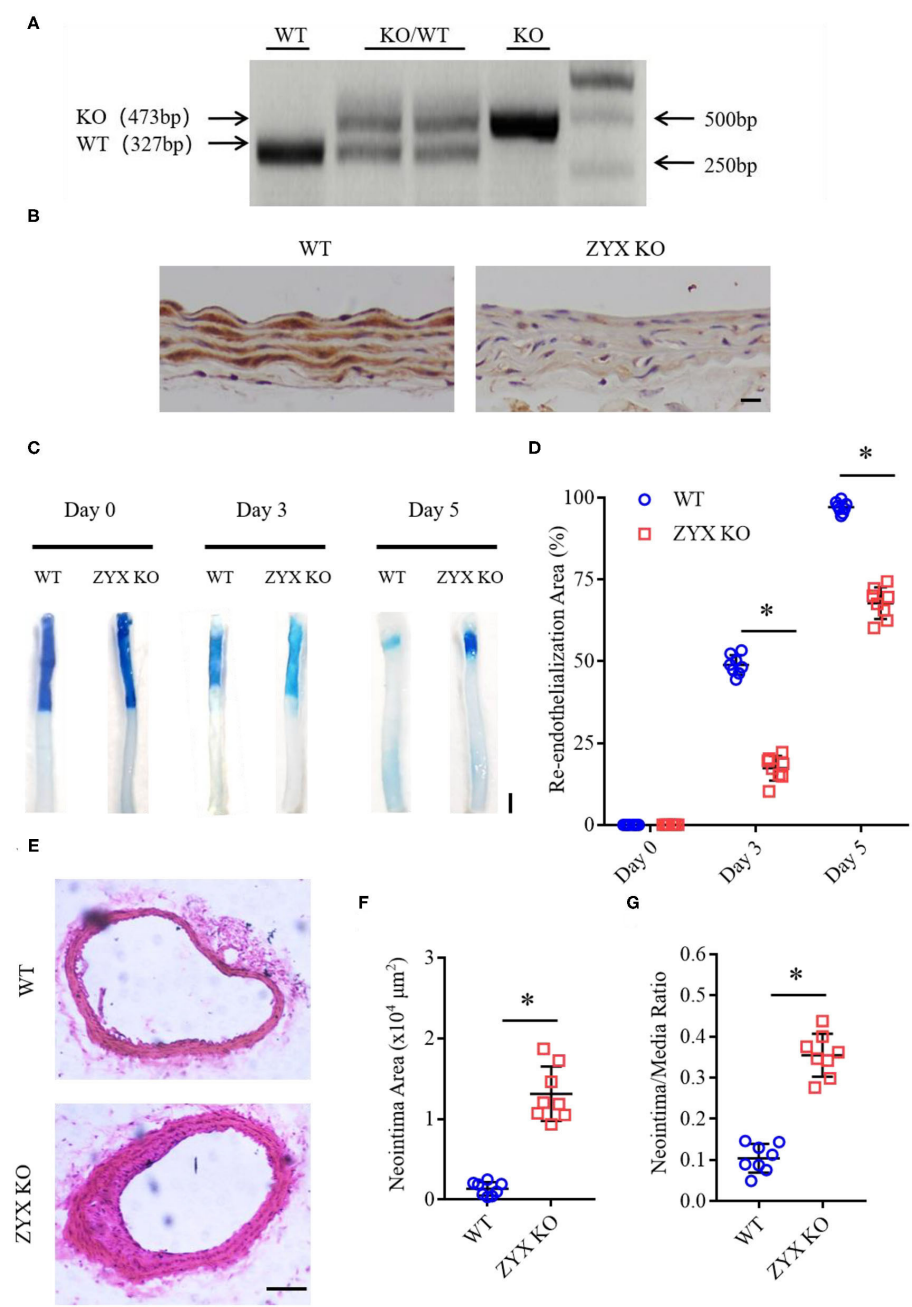
Zyxin deficiency delays re-endothelialization and promotes neointima formation in wire-injured mice carotid arteries. **(A)** Results of genotyping polymerase chain reaction (PCR) for WT and ZYX KO mice (KO for 473 bp, WT for 327 bp). **(B)** Immunohistochemistry (IHC) staining of zyxin in the WT and ZYX KO mice (scale bar, 10 μm). **(C)** Representative image of re-endothelialization assessed by Evans blue staining showing endothelial denudation of carotid arteries 0, 3, and 5 days after injury (scale bar, 1 mm). **(D)** Quantification of the re-endothelialization area after injury. **(E)** Neointima formation was determined using cross-sections of carotid arteries by hematoxylin and eosin (HE) staining 28 days after injury (scale bar, 100 μm). **(F)** Quantification of neointima formation area. **(G)** Quantification of neointima/media ratio. Mean ± standard deviation (SD), *n* = 8, * *P* < 0.05. Test of Normality: *P* > 0.05, a test of homogeneity: *P* > 0.05. WT, wild-type; ZYX KO, zyxin knockout.

Carotid artery wire injury is widely used for studies on both endothelial post-injury recovery and post-angioplasty restenosis, as it closely resembles the angioplasty procedure that injures both the endothelial layer and the vessel wall (Lindner et al., 1993). The procedure denudes the endothelium and triggers a repair process that reaches completion around 1 week after the injury. To determine the role of zyxin in the regulation of re-endothelialization after vascular injury, carotid arteries from conventional zyxin knockout mice (ZYX KO) and wild type (WT) controls (Figures 1A,B) were injured with a guidewire (at 5 mm, Supplementary Figure 1), and the mice were euthanized 3 or 5 days after the injury to evaluate the progress of endothelial repair using Evans blue dye. As shown in Figure 1C, at day 5 after injury, the endothelial coverage of the luminal lining as indicated by the dye-free area of denuded arterial segments was significantly less in the arteries from ZYX KO mice than in the controls (Figure 1D). We also noticed that zyxin deficiency significantly increased neointima hyperplasia compared with wild-type mice at day 28 after injury (Figures 1E–G).

**Figure 2:**
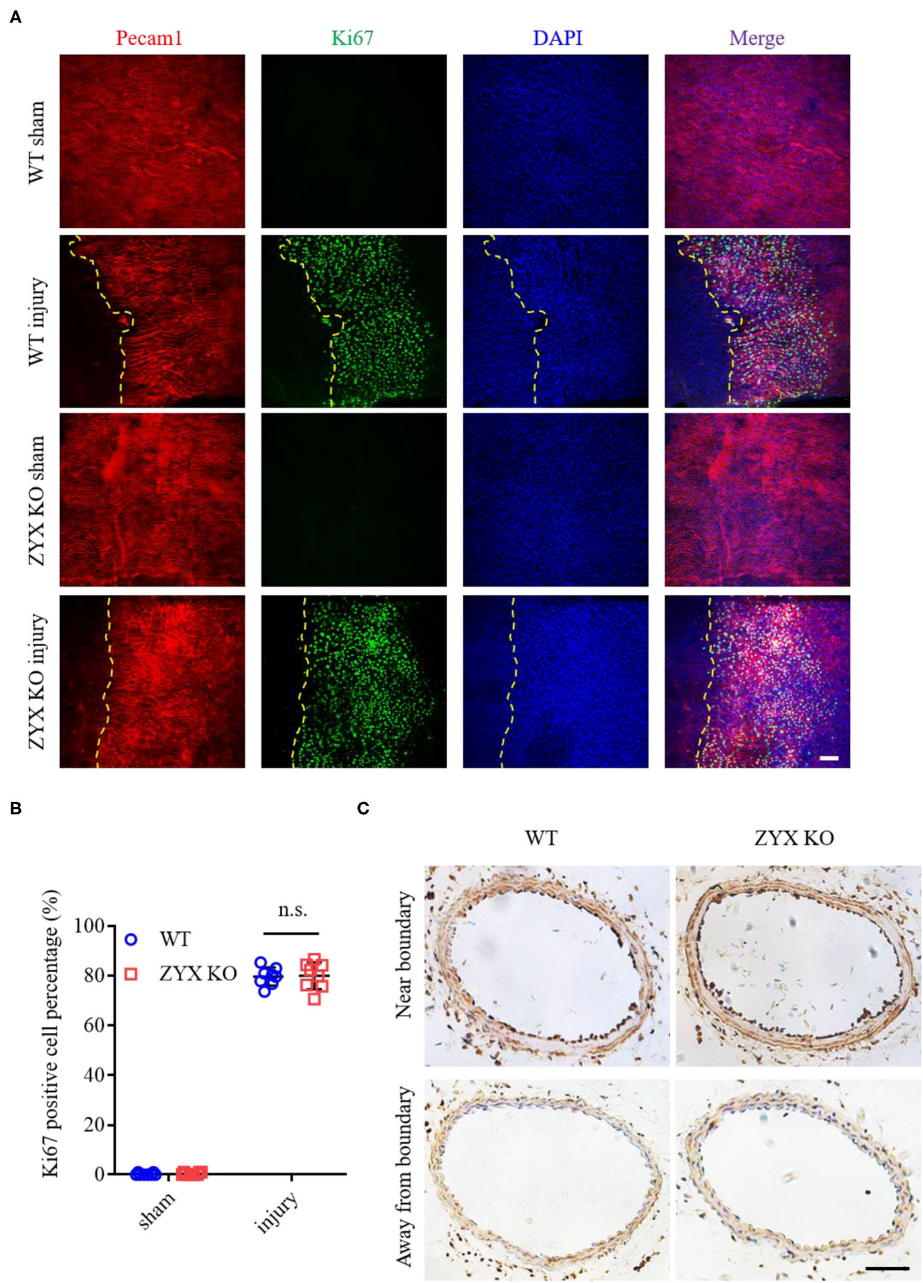
Proliferation is not involved in the re-endothelialization of injured arteries in zyxin-deficient mice. **(A)** Representative immunofluorescence staining of Pecam1, Ki67, and 4', 6-diamidino-2 phenylindole dihydrochloride (DAPI) in carotid arteries 3 days after injury (scale bar, 25 μm). **(B)** Quantification of the Ki67 positive cell percentage. **(C)** Representative IHC staining of Ki67 in carotid artery cross-sections that are near (upper) or away (below) from the injured boundary 3 days after injury (scale bar, 100 μm). Mean ± SD, *n* = 8; n.s., not significantly different. Test of Normality: *P* > 0.05, test of homogeneity: *P* > 0.05. WT, wild type; ZYX KO, zyxin knockout.

Since the re-endothelialization of injured vessels involves endothelial proliferation and migration, we then measured the proliferation rate of ECs in injured and sham-operated arteries by immunostaining Pecam1 (an EC marker) and Ki67 (a proliferation marker). Pecam1/Ki67 double-positive cells were rarely observed in sham-operated arteries. In contrast, the number of Ki67-positive ECs increased dramatically in injured arteries from the ZYX KO mice in a manner similar to injured arteries from the control mice (Figures 2A,B). Besides, endothelial proliferation occurs more near the injured boundary than away from the boundary (Figure 2C). These results suggest that zyxin mediates vascular repair after artery injury likely *via* endothelial migration.

### Zyxin Deletion in ECs Impairs the Re-endothelialization of Injured Mouse Arteries

**Figure 3:**
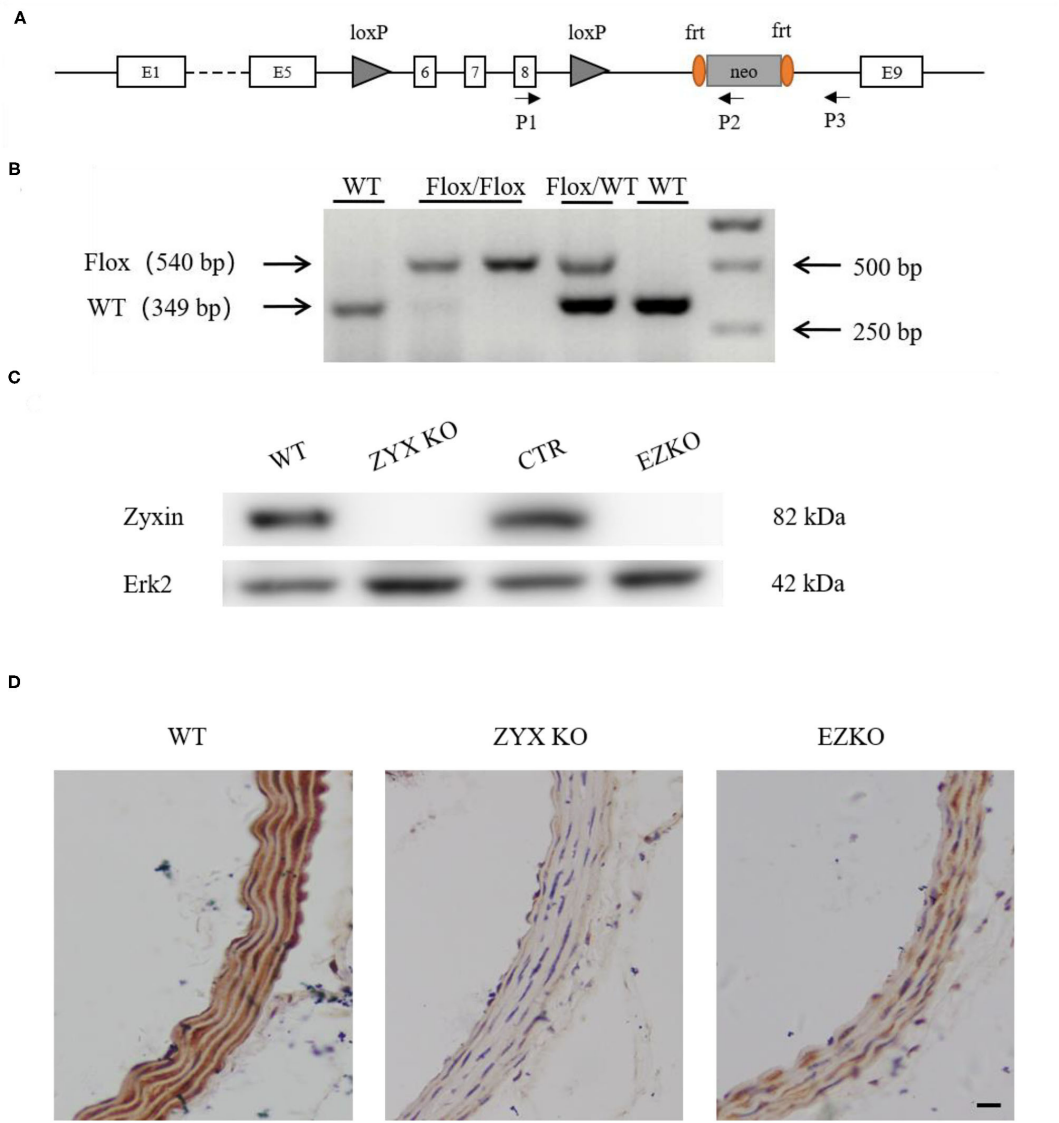
Generation of endothelium-specific zyxin knockout mice. **(A)** Construction strategy for the *Zyx*^*flox*/*flox*^ mice. Loxp sites are inserted in the introns after exons 5 and 8 of zyxin. **(B)** Results of genotyping PCR, floxed for 540 bp, WT for 349 bp. **(C)** Western blots of protein levels of zyxin and Erk2 in WT, ZYX KO, CTR, and EZKO mouse brain vascular endothelial cells. **(D)** IHC staining of zyxin in the WT, ZYX KO, and EZKO mice (scale bar, 10 μm). WT, wild-type; ZYX KO, zyxin knockout; CTR, control; *Zyx*^*flox*/*flox*^; *Tie 2-Cre/*-; EZKO, endothelium-specific zyxin knockout, *Zyx*^*flox*/*flox*^; *Tie 2-Cre/*+.

**Figure 4:**
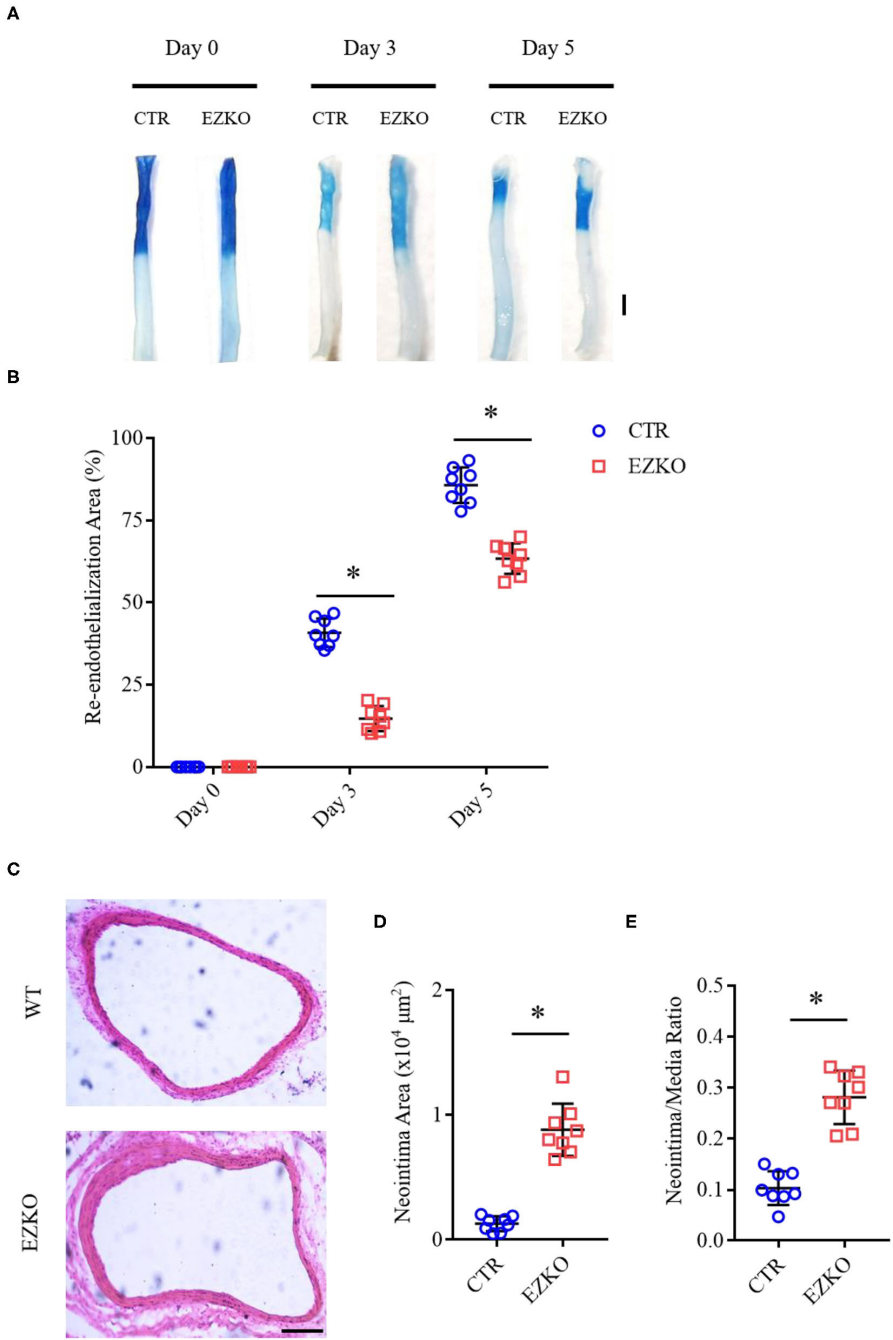
Zyxin deletion in ECs impairs the re-endothelialization and promotes neointima formation in wire-injured mouse carotid arteries. **(A)** Representative image of re-endothelialization assessed by Evans blue staining showing endothelial denudation of carotid arteries 0, 3, and 5 days after injury (scale bar, 1 mm). **(B)** Quantification of the re-endothelialization area after injury. **(C)** Neointima formation was determined using cross-sections of carotid arteries by HE staining 28 days after injury (scale bar, 100 μm). **(D)** Quantification of neointima formation area. **(E)** Quantification of neointima/media ratio. Mean ± SD, *n* = 8, *P < 0.05. Test of normality: *P* > 0.05, test of homogeneity: *P* > 0.05. CTR, control; EZKO, endothelium-specific zyxin knockout.

Zyxin is expressed in multiple cell types, such as fibroblasts (Hoffman et al., 2006), vascular smooth muscle cells (Cattaruzza et al., 2004), and ECs (Han et al., 2017). To determine whether zyxin deficiency in ECs contributes to the phenotype of impaired vascular repair in the injured carotid arteries of the ZYX KO mice after injury, endothelium-specific zyxin knockout (EZKO) mice were established. They were generated by crossing a transgenic mouse line expressing Cre under the control of a Tie2 endothelium-specific promoter (Lan et al., 2007) with mice carrying a floxed zyxin allele (Figures 3A,B). An immunoblot analysis demonstrated that zyxin protein expression was decreased by ~95% in ECs isolated from the brain tissue of the EZKO mice, compared with those from the control mice (Figure 3C). It should be noted here that Erk2 is used as a loading control, which may be better than conventional controls such as tubulin, as shown in Supplementary Figure 4 and our previous articles (Han et al., 2017). An immunohistochemistry analysis shows endothelial-specific deletion of zyxin in common carotid artery tissue (Figure 3D). As shown in Figure 4, the EZKO mice exhibit a significantly impaired endothelial recovery. The endothelial coverage in the injured carotid arteries of EZKO mice was significantly less than controls (Figures 4A,B), suggesting that endothelial zyxin is required for vascular repair. Besides, we also noticed that the endothelium-specific zyxin knockout (EZKO) mice show a significant increase in neointima hyperplasia compared with the control mice (Figures 4C–E).

### Zyxin Deletion Reduces Endothelial Migration in Primary Cultured Human ECs

**Figure 5:**
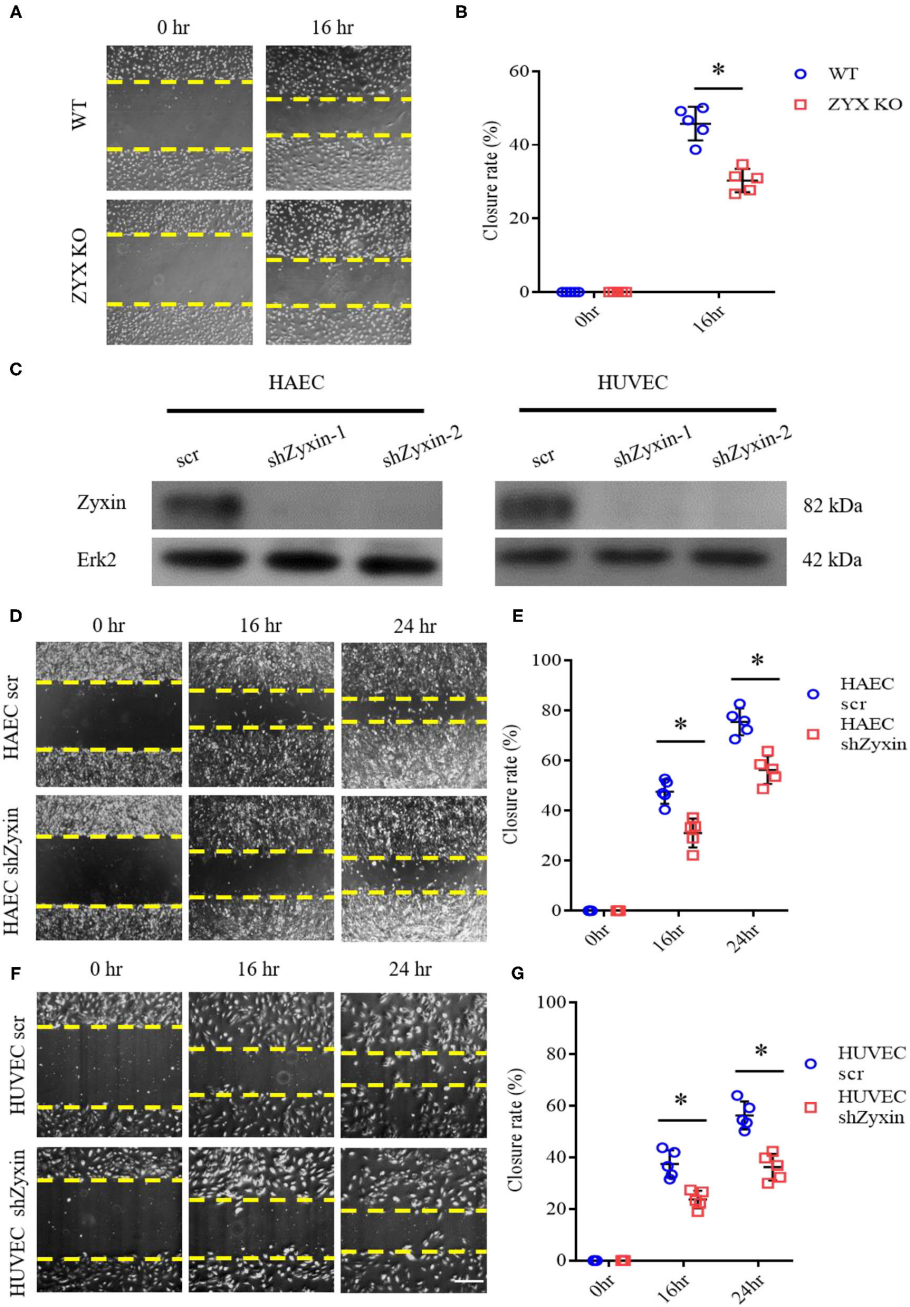
Zyxin deficiency reduces vascular endothelial cell migration. **(A)** Migration of primary ECs isolated from the WT and ZYX KO mice was assessed by a scratching wound-healing assay. Representative images were taken 0 and 16 h post scratching. **(B)** Quantification of mean closure rate 16 h after scratching. **(C)** Western blots of protein level of zyxin and Erk2 in human amniotic epithelial cells (HAECs) and human umbilical vein endothelial cells (HUVECs) expressing scrambled shRNA (scr) or Zyxin shRNAs (shZyxin-1, shZyxin-2). **(D,F)** Representative images of the effect of zyxin on the migration of HAECs and HUVECs assessed by a scratching wound-healing assay at 0, 16, and 24 h post scratching (scale bar, 100 μm). **(E,G)** Quantification of mean closure rate 16 and 24 h after scratching. Mean ± SD, *n* = 5, *P < 0.05. Test of normality: *P* > 0.05, test of homogeneity: *P* > 0.05.

To directly address the effect of zyxin on EC migration, we used an *in vitro* model of cell wound healing in which an incision-like gap was made across confluent monolayers of the ECs. The “wounded” area was photographed immediately after scratching at the indicated time points, and cell migration was quantified and expressed as the average percentage of closure rate of the scratch area. To exclude the involvement of cell proliferation, the ECs were starved by serum deprivation or treated with mitomycin C, a proliferation inhibitor. As shown in Supplementary Figure 2, these experimental conditions display similar results. We first compared the capabilities of primary ECs isolated from the WT and ZYX KO mice and found that the ZYX KO mice-derived ECs migrated significantly slower than the WT cells (Figures 5A,B). Then, we employed primary human ECs expressing zyxin-specific shRNAs (Han et al., 2017), which had significant knockdown efficacy (Figure 5C). The ECs expressing shzyxin migrated significantly more slowly than the controls (Figures 5D–G), suggesting that zyxin mediates cell migration during endothelial repair in the wound-healing process. Finally, we measured the cell proliferation rate directly and found that zyxin deficiency had no effect on cell proliferation (Supplementary Figure 3), further confirming its major role in endothelial migration.

### Zyxin Is Required for Forskolin-Induced Endothelial Migration in Primary Cultured Human ECs

**Figure 6:**
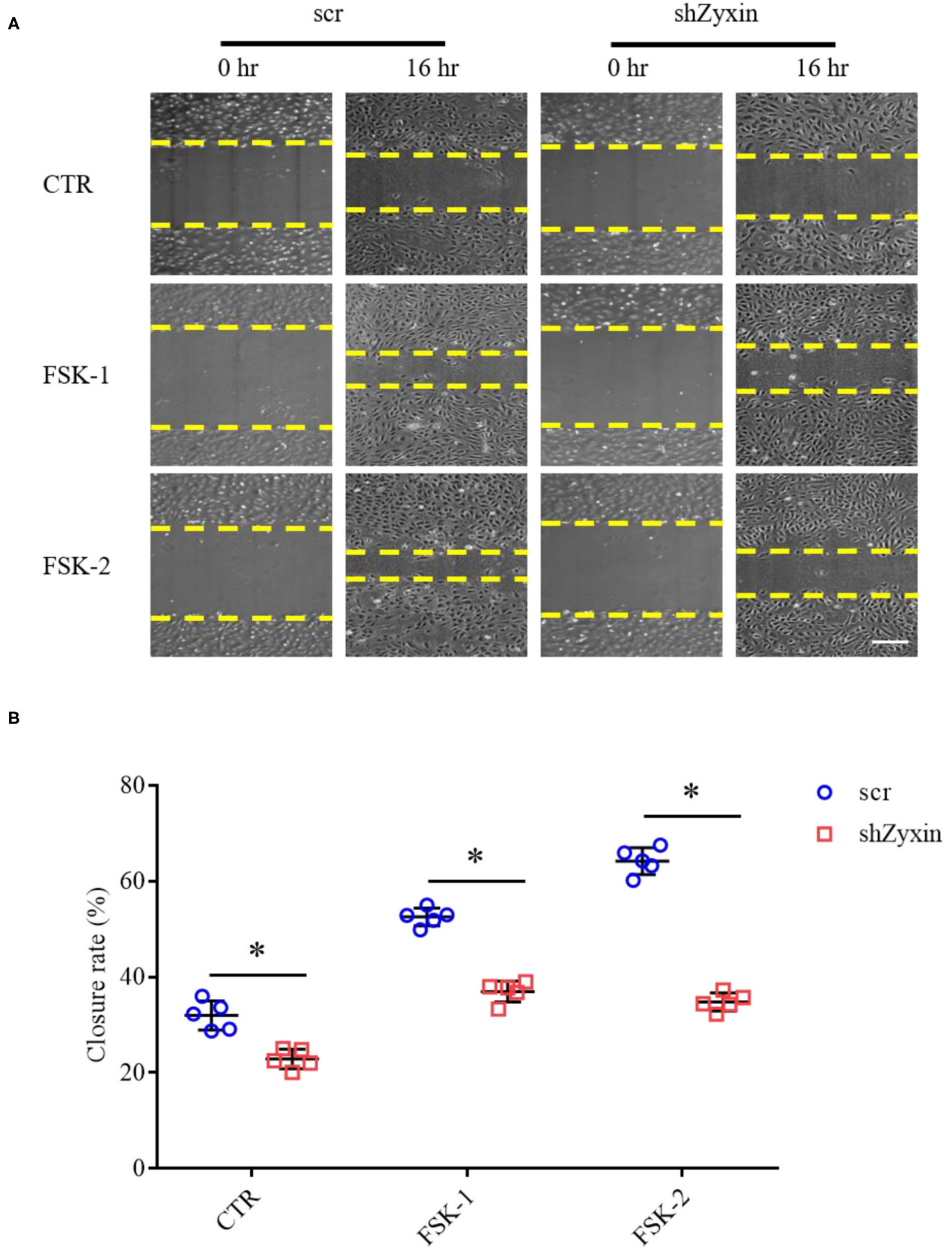
Zyxin deletion in HUVECs decreases migration under forskolin stimulation. **(A)** Representative images of the effect of zyxin on HUVEC migration under forskolin (FSK) stimulation assessed by a scratching wound-healing assay 0 and 16 h post scratching (scale bar, 500 μm). HUVECs expressing scrambled shRNA (scr) or zyxin shRNAs (shZyxin) were stimulated with two concentrations of forskolin (FSK-1: 1 μM, FSK-2: 5 μM) or vehicle (CTR). **(B)** Quantification of mean closure rate 16 h after scratching. Mean ± SD, *n* = 5, *P < 0.05. Test of normality: *P* > 0.05, test of homogeneity: *P* > 0.05.

**Figure 7:**
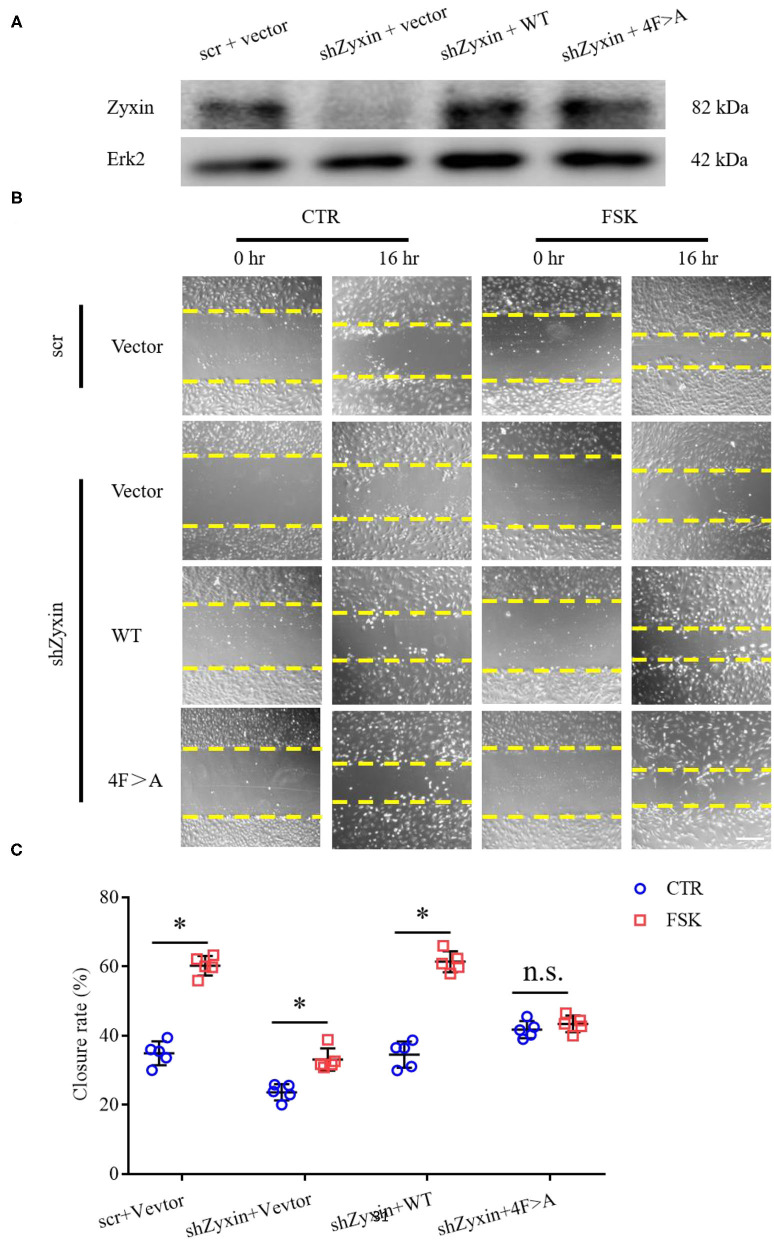
Decreased migration under cAMP pathway stimulation is vasodilator-stimulated phosphoprotein (VASP)-independent in zyxin deleted HUVECs. **(A)** Western blots of protein levels of both zyxin and the zyxin 4F>A mutant, and Erk2 in HUVECs. **(B)** Representative images of the effect of zyxin on HUVEC migration under cAMP pathway stimulation in the presence of forskolin (FSK: 5 μM) or vehicle (CTR) assessed by scratching wound-healing assay 0 h and 16 h post scratching (scale bar, 500 μm). **(C)** Quantification of mean closure rate 16 h after scratching. Mean ± SD, *n* = 5, *P < 0.05, n.s., not significantly different. Test of normality: *P* > 0.05, test of homogeneity: *P* > 0.05.

Our recent study showed that zyxin is critical for cAMP agonist-induced von Willebrand factor (VWF) secretion in cultured primary human ECs and mouse models of hemostasis and thrombosis, suggesting a role of zyxin-mediated cAMP signaling in maintaining blood vessel homeostasis (Han et al., 2017). We, thus, studied the possible involvement of zyxin in cell migration induced by forskolin (a well-known cAMP agonist). As shown in Figure 6, forskolin is capable of stimulating endothelial migration, which is inhibited when zyxin is downregulated. Vasodilator-stimulated phosphoprotein (VASP) is an important binding partner of zyxin-mediated cell migration (Smith et al., 2010; Sperry et al., 2010). Using a zyxin mutant deficient in binding VASP (Han et al., 2017), we found that ECs expressing VASP-binding mutation failed to respond to forskolin stimulation, albeit there was a slight increase in the basal level of migration (Figure 7). These data indicate that zyxin mediates forskolin-induced migration in a VASP-dependent manner.

### Zyxin Is Required for Forskolin-Induced Re-endothelialization of Injured Mouse Arteries

**Figure 8:**
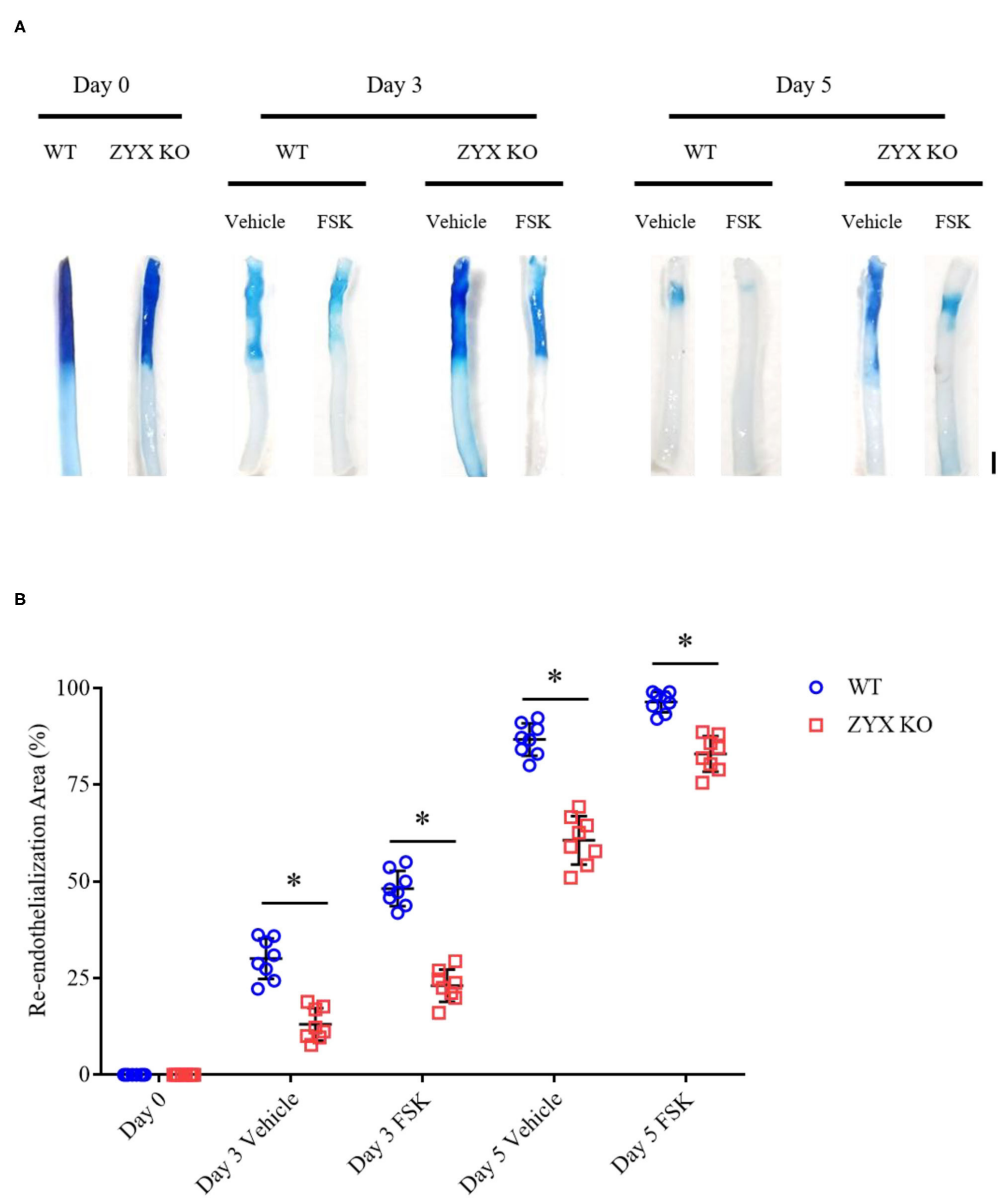
Zyxin deficiency compromises re-endothelialization in injured arteries under forskolin stimulation. **(A)** Representative images of endothelial denudation assessed by Evans blue staining of carotid arteries 0, 3, and 5 days after injury with forskolin (FSK: 10 mg kg^−1^, daily injection) or vehicle treatment (scale bar, 1 mm). **(B)** Quantification of the re-endothelialization area after injury. Mean ± SD, *n* = 8, *P < 0.05. Test of normality: *P* > 0.05, test of homogeneity: *P* > 0.05. WT, wild-type; ZYX KO, zyxin knockout.

**Figure 9:**
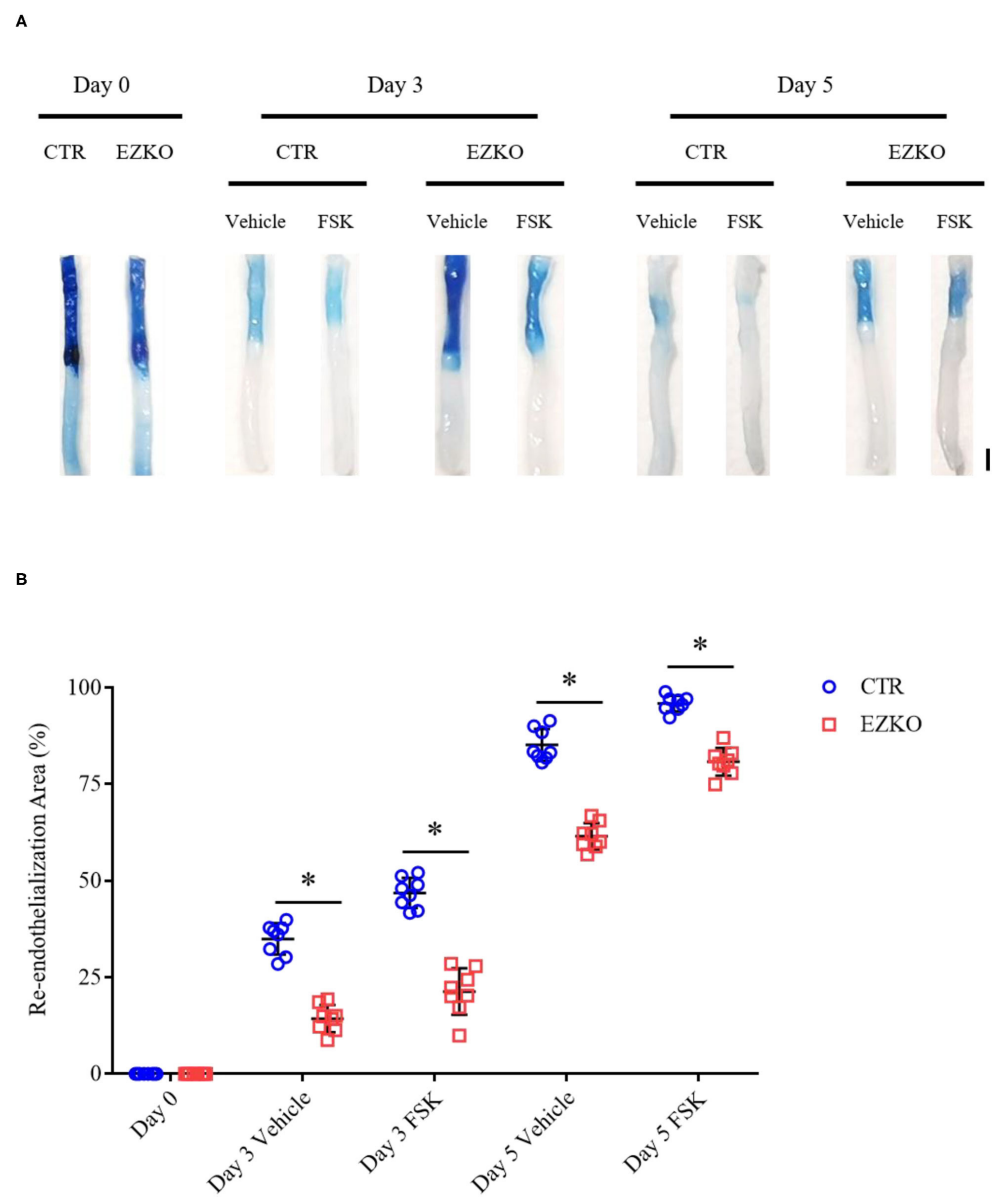
Endothelium-specific deletion of zyxin compromises re-endothelialization in injured arteries under forskolin stimulation. **(A)** Representative images of endothelial denudation assessed by Evans Blue staining of carotid arteries 0, 3, and 5 days after injury with forskolin (FSK: 10 mg kg^−1^, daily injection) or vehicle treatment (scale bar, 1 mm). **(B)** Quantification of the re-endothelialization area after injury. Mean ± SD, *n* = 8, *P < 0.05. Test of normality: *P* > 0.05, test of homogeneity: *P* > 0.05. WT, wild-type; EZKO, endothelium-specific zyxin knockout; FSK, forskolin.

Based on the results of *in vitro* wound-healing assays and early preclinical and clinical data that suggest a potential role of forskolin in treating cardiovascular diseases (Bhat et al., 1983; Baumann et al., 1990), we determined further whether forskolin has a specific effect on post-injury vascular repair and the possible involvement of zyxin in the effect, if any. In agreement with the results of *in vitro* cellular wound-healing assays, the injured arteries from mice that received forskolin treatment recovered more quickly than those that received solvent control. In addition, the forskolin-stimulated endothelial repair was significantly compromised in zyxin-deficient mice, both ZYX KO (Figure 8) and EZKO mice (Figure 9).

These results indicate that forskolin is capable of promoting the vascular repair of injured carotid arteries and that this is, at least in part, mediated by endothelial zyxin.

## Discussion

A thorough understanding of intracellular signaling components and pathways leading to re-endothelialization would be instrumental in designing new strategies for repairing injured vessels. Here, we present several lines of evidence supporting an essential role of zyxin-mediated signaling in vascular repair and its therapeutic potential as a target. The critical function of zyxin in re-endothelialization was first shown by the phenotype of impaired vascular recovery after wire injury in mice with complete knockout (Figure 1). The indispensable function of endothelial zyxin in post-injury vascular repair was shown further by the similar phenotype in mice with endothelium-specific knockout (Figure 4). The important function of zyxin in ECs was directly demonstrated using cultured primary human HUVECs and HAECs expressing zyxin shRNAs (Figure 5) in a wound-healing assay. Mechanistically, zyxin mediates signaling in the regulation of endothelial migration, but not cell proliferation, and this is, depending on its interaction with VASP (Figure 7), a well-known partner of zyxin in modulating cellular actin remodeling.

In the literature, zyxin regulates cell migration likely in a cell-context dependent manner. In an early study, Hoffman et al. characterized the phenotype of fibroblasts in which the zyxin gene was deleted by homologous recombination and showed that zyxin-null fibroblasts display enhanced integrin-dependent adhesion and are more migratory than wild-type fibroblasts (Hoffman et al., 2006). In another study on *Drosophila* hemocytes by Moreira et al., zyxin depletion in hemocytes leads to a significant increase in cell speed without affecting their response to a chemotactic cue (Moreira et al., 2013). In contrast, Choi et al. showed that renal epithelial with zyxin knockdown have slower wound healing than control cells transfected with siRNA, and that wound healing is not accelerated by epidermal growth factor (EGF) (Choi et al., 2013). Similarly, in breast epithelial cells, Ma et al. reported that depletion of zyxin impairs the capacity for cell migration (Ma et al., 2016). Future studies are needed to investigate the intracellular signaling details and cytoskeletal changes in order to understand the discrepancy in the differential roles of zyxin in the control of cell movement.

In a recent study, we showed that zyxin is an important regulator of cAMP agonist-induced VWF secretion in cultured primary human ECs and mouse models of hemostasis and thrombosis, suggesting a crucial role of zyxin-mediated cAMP signaling in maintaining blood vessel homeostasis (Han et al., 2017). In this study, we further revealed zyxin as a regulator of post-injury vascular repair *via* mediating endothelial migration, which was promoted by forskolin treatment (Figure 8). A natural product isolated from the Indian plant *Coleus forskohlii*, forskolin has been shown to be effective in lowering blood pressure in rats, cats, and pigs (Bhat et al., 1983). Notably, in 12 patients with stage III (NYHA) congestive cardiomyopathy, forskolin dose-dependently reduced cardiac pre- and afterload values, and led to a reduction in systolic, diastolic, and mean pulmonary artery pressures as well as pulmonary wedge pressure by >50% concomitant with an increase in cardiac output (Baumann et al., 1990). Consistent with a recent report (Hao et al., 2020) from Hao et al., this study demonstrates the ability of forskolin to promote endothelial recovery from vascular injury and indicates its therapeutic applications in disorders associated with vascular injuries, such as in-stent restenosis after treatment for coronary stenosis.

In summary, our study not only identifies zyxin to be a critical regulator of endothelial repair but also demonstrates the therapeutic potentials of zyxin-mediated signaling and its agonist forskolin in treating disorders associated with vascular injury.

## Data Availability Statement

The raw data supporting the conclusions of this article will be made available by the authors, without undue reservation.

## Ethics Statement

The animal study was reviewed and approved by the Center for Experimental Animals (a facility accredited by the Association for Assessment and Accreditation of Laboratory Animal Care) at Peking University, Beijing, China.

## Author Contributions

JL designed the experiments and wrote the manuscript. XK, YD, YC, and YH carried out the experiments. XK analyzed the data and drafted the manuscript. All authors contributed to the article and approved the submitted version.

## Funding

This study was supported by the National Natural Science Foundation of China (81930011, 31771263, and 91739304), the National Key R&D Program of China (2019YFA0801603), and the Peking University Clinical Scientist Program (BMU2019LCKXJ001) from the Fundamental Research Funds for Central Universities.

## Conflict of Interest

The authors declare that the research was conducted in the absence of any commercial or financial relationships that could be construed as a potential conflict of interest.

## Publisher's Note

All claims expressed in this article are solely those of the authors and do not necessarily represent those of their affiliated organizations, or those of the publisher, the editors and the reviewers. Any product that may be evaluated in this article, or claim that may be made by its manufacturer, is not guaranteed or endorsed by the publisher.
