# Supplementary material for: Zyxin Mediates Vascular Repair via Endothelial Migration Promoted by Forskolin in Mice
Source: Front Physiol. 2021 Oct 8;12:741699. doi: 10.3389/fphys.2021.741699 (PMC8531502; doi:10.3389/fphys.2021.741699)
Supplement: Supplementary file 1 [file Data_Sheet_1.pdf]

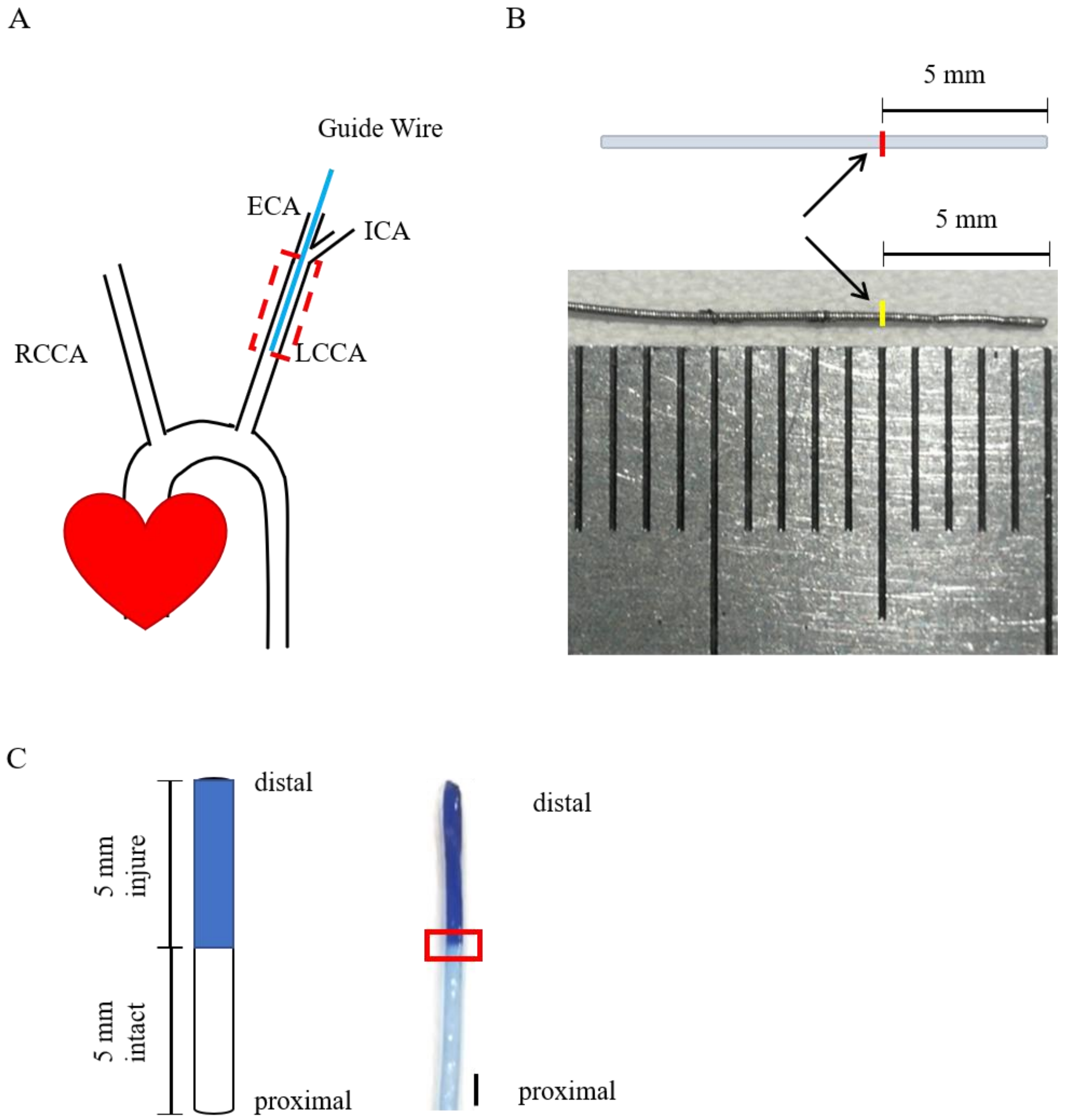

Supplemental Figure 1. Overview of the CCA wire injury model. (A) Cartoon of the CCA wire injury model outline (blue line, guide wire; red dashed line, the injury site of the LCCA). LCCA, left common carotid artery; RCCA, right common carotid artery; ECA, external carotid artery; ICA, internal carotid artery. (B) Cartoon (above) and representative image (below) indicating the injury depth by a guide wire. (C) Cartoon (left) and representative image (right) of a carotid artery stained with Evans Blue on the day of injury (red box, site for morphological analysis in whole-mount staining; scale bar: 1 mm).

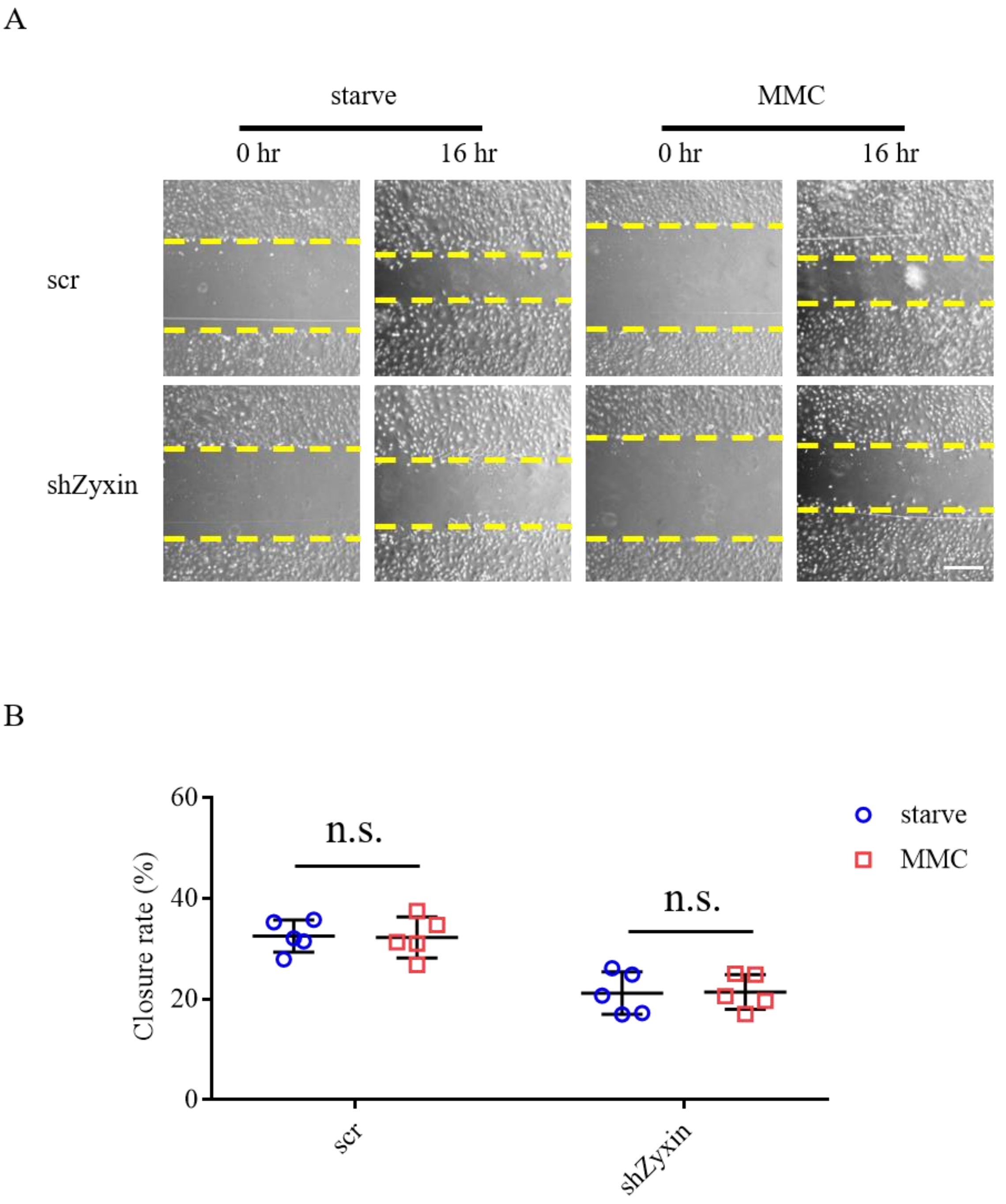

Supplemental Figure 2. Starvation and mitomycin C treatment shows same inhibition effect of EC proliferation during migration. (A) The effect of starvation and mitomycin C (MMC) treatment inhabitation proliferation during migration was assessed by a scratching wound-healing assay. HUVECs expressing scrambled shRNA (scr) or Zyxin shRNAs (shZyxin) were treated with 6% FBS or 5μg/ml MMC. Representative images were taken at 0 hr and 16 hr post scratching (scale bar, 500 μm). (B) Quantification of the mean closure rate 16 hr after scratching. Data are mean ± SD, n=5. n.s., not significantly different. Test of Normality: $P > 0.05$，test of Homogeneity: $P > 0.05$.

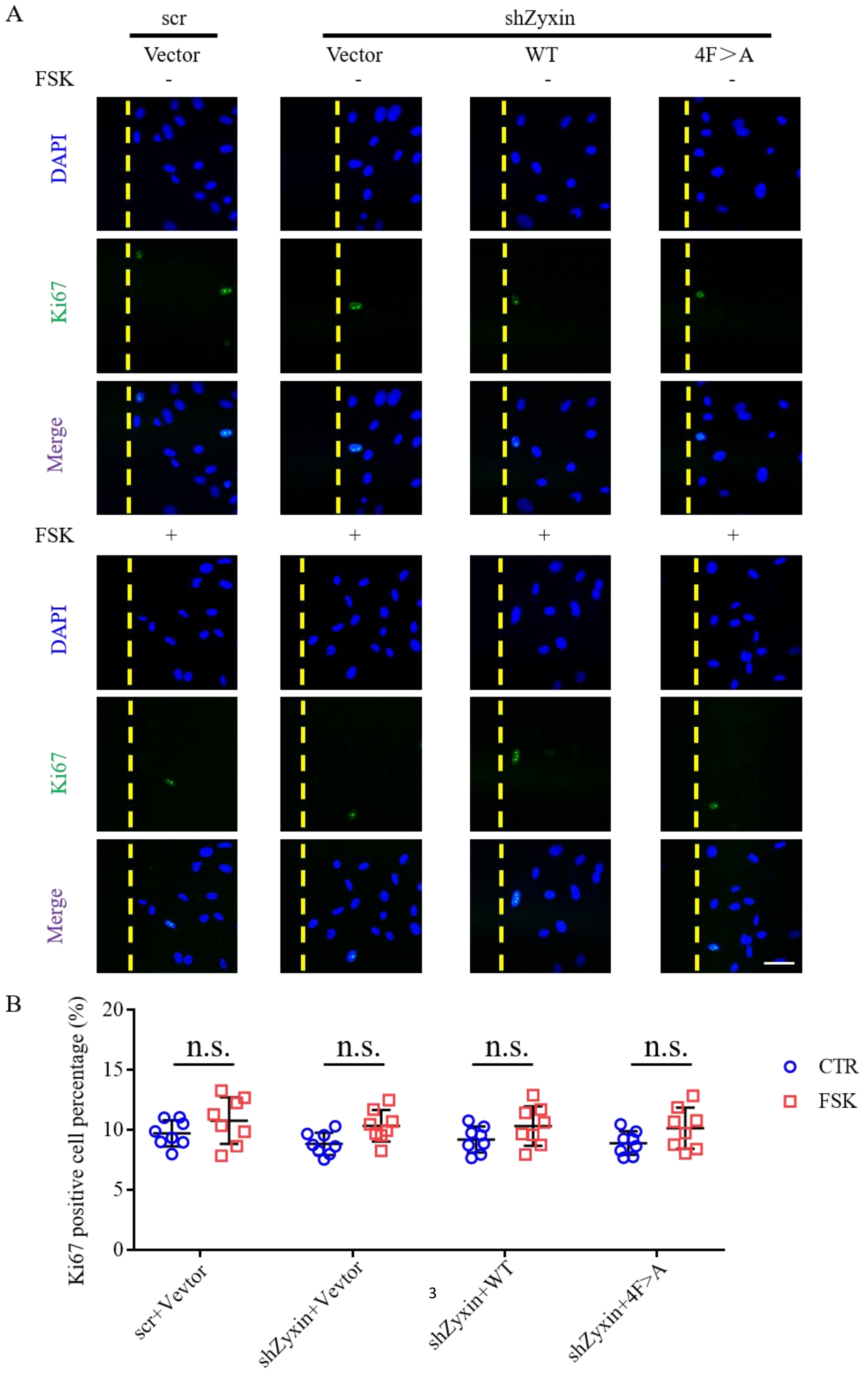
A
scr
shZyxin
Vector
Vector
WT
4F>A
FSK
-
-
-
-
DAPI
Ki67
Merge
FSK
+
+
+
+
DAPI
Ki67
Merge
B
Ki67 positive cell percentage (%)
20
15
10
5
0
n.s.
n.s.
n.s.
n.s.
CTR
FSK
scr+Vevtor
shZyxin+Vevtor
shZyxin+WT
shZyxin+4F>A

Supplemental Figure 3. Proliferation is not involved in migration of ECs with zyxin deficiency under FSK treatment. (A) Proliferation was measured by IF staining of ki67 at 16hr after scratching. Representative immunofluorescence staining of Ki67 and DAPI in HUVECs during migration (scale bar, 10 μm). (B) Quantification of the Ki67 positive cell percentage. Data are mean ± SD, n=8. n.s., not significantly different. Test of Normality: P > 0.05， test of Homogeneity: P > 0.05.

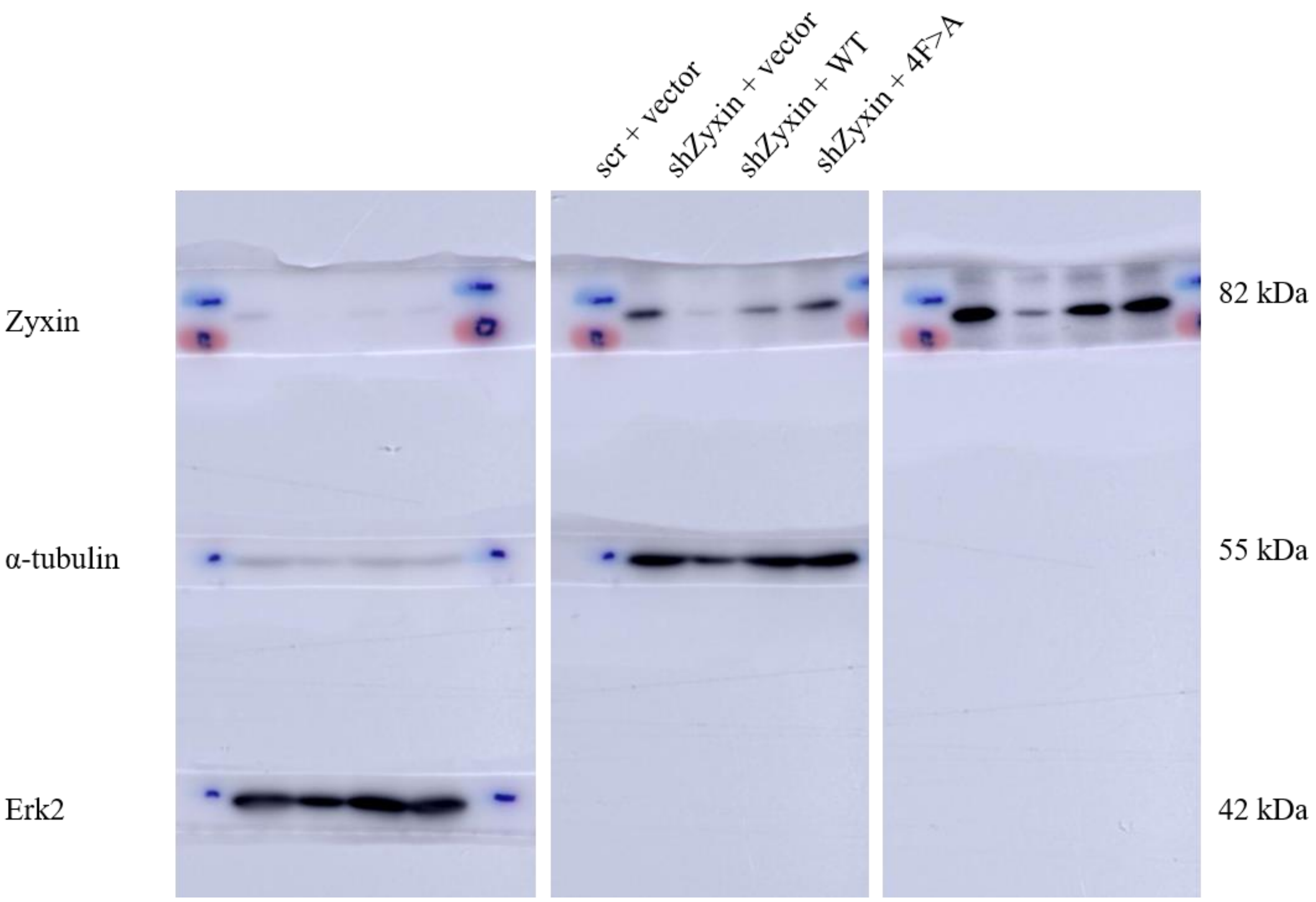

Supplemental Figure 4. Western blots of zyxin with α-tubulin or Erk2 as a loading control. Western blots (with different exposure times of chemiluminescence: left, 0.5 s; middle, 1.0 s; right, 2.5 s), which are presented with at least 2 flacking ladders, show protein levels of both zyxin, α-tubulin, and Erk2 in HUVECs.

.
